# Immunogenicity of Seven-Valent Pneumococcal Conjugate Vaccine Administered at 6, 14 and 40 Weeks of Age in South African Infants

**DOI:** 10.1371/journal.pone.0072794

**Published:** 2013-08-28

**Authors:** Stephanie A. Jones, Michelle Groome, Anthonet Koen, Nadia Van Niekerk, Poonam Sewraj, Locadiah Kuwanda, Alane Izu, Peter V. Adrian, Shabir A. Madhi

**Affiliations:** 1 Department of Science/National Research Foundation: Vaccine Preventable Diseases, University of the Witwatersrand, Faculty of Health Science, Johannesburg, South Africa; 2 Medical Research Council, Respiratory and Meningeal Pathogens Research Unit, Johannesburg, South Africa; 3 National Institute for Communicable Diseases-a division of National Health Laboratory Service, Centre for Vaccines and Immunology, Sandringham, South Africa; Centers for Disease Control & Prevention, United States of America

## Abstract

**Background:**

The high cost of pneumococcal conjugate vaccine (PCV) and local epidemiological factors contributed to evaluating different PCV dosing-schedules. This study evaluated the immunogenicity of seven-valent PCV (PCV7) administered at 6-weeks; 14-weeks and 9-months of age.

**Methods:**

250 healthy, HIV-unexposed infants were immunized with PCV7 concurrently with other childhood vaccines. Serotype-specific anti-capsular IgG concentrations were measured one-month following the 1^st^ and 2^nd^ PCV-doses, prior to and two-weeks following the 3^rd^ dose. Opsonophagocytic killing assay (OPA) was measured for three serotypes following the 2^nd^ and 3^rd^ PCV7-doses. Immunogenicity of the current schedule was compared to a historical cohort of infants who received PCV7 at 6, 10 and 14 weeks of age.

**Results:**

The proportion of infants with serotype-specific antibody ≥0.35 µg/ml following the 2^nd^ PCV7-dose ranged from 84% for 6B to ≥89% for other serotypes. Robust antibody responses were observed following the 3^rd^ dose. The proportion of children with OPA ≥8 for serotypes 9V, 19F and 23F increased significantly following the 3^rd^ PCV7-dose to 93.6%; 86.0% and 89.7% respectively. The quantitative antibody concentrations following the 2^nd^ PCV7-dose were comparable to that after the 3^rd^ -dose in the 6-10-14 week schedule. Geometric mean concentrations (GMCs) following the 3^rd^ PCV7-dose were higher for all serotypes in this study compared to the historical cohort.

**Conclusions:**

The studied PCV7 dosing schedule induced good immune responses, including higher GMCs following the 3^rd-^dose at 9-months compared to when given at 14-weeks of age. This may confer longer persistence of antibodies and duration of protection against pneumococcal disease.

## Introduction

The World Health Organization recommends that 10- or 13-valent pneumococcal conjugate vaccine (PCV) be introduced into immunization programs either as a three dose primary series (3+0) or two doses during infancy followed by a third dose in the second-year of life (2+1 schedule) [1]. The choice between these two schedules should include consideration of local epidemiological factors [1]. A meta-analysis of three compared to a two-dose primary series of PCV7 during infancy, indicated similar immunogenicity to the majority of serotypes between the two dosing-schedules [2,3], except for serotypes 6B and 23Ffor which antibody geometric mean concentrations (GMC’s) and the proportion of infants with serotype-specific antibody concentrations ≥0.35 µg/ml were lower following a 2-dose compared to after a 3-dose primary series [2,3]. Additionally, the immunogenicity following two doses of PCV in infants is enhanced by spacing the doses two-months compared to one-month apart [4].

The high cost of PCV, has been a major factor for adopting vaccine schedules requiring fewer doses than a 3+1 schedule, which was used in the pivotal study upon which PCV7 (Prevenar®) was licensed [5]. Elsewhere, non-licensure studies in Africa established vaccine efficacy against invasive pneumococcal disease and pneumonia with a three dose primary-series only [6,7]. The absence of a booster dose in the South African study was, however, associated with waning of protection in anti-retroviral naïve HIV-infected children [8]. HIV-infected children contribute to approximately 55% of all invasive pneumococcal disease in South Africa despite access to antiretroviral treatment and have a >40-fold heightened risk of developing disease beyond two-years of age [9]. PCV7 was introduced into the South African immunization program since April 2009, at a unique schedule of 6 and 14 weeks of age, followed by a third dose at approximately 9-months of age. The rational for this schedule included cost-effectiveness considerations of a four-dose schedule, as well as aiming at extending the persistence of protection in vulnerable groups such as HIV-infected children. The choice of providing the third dose at 9-months of age, was based on children already being scheduled to receive their first dose of measles-vaccine at this age, coupled with a higher uptake of the first measles-dose compared to the second-dose which is administered at 15-18 months of age. The immunogenicity of the PCV-dosing schedule adopted in South Africa had not been evaluated.

The aim of this study was to determine the immunogenicity of the novel PCV dosing schedule given at 6-weeks, 14 weeks and 9-months of age. Also, we compared the immune responses of the latter schedule to that of a historical cohort who had received PCV7 at 6, 10 and 14 weeks of age as described [10,11].

## Methods

### Study population and study-design

250 healthy infants, born to HIV-uninfected mother, were enrolled between October 2009 and February 2010 in a prospective, longitudinal cohort study. Potential study-participants were identified through the birth-registry at Chris Hani Baragwanath Academic Hospital (CHBAH, Soweto, Johannesburg) and at the well-baby, immunization clinic at an adjacent primary health care clinic (Diepkloof Clinic). Healthy 6-8 week old infants, born at term to mothers documented as being HIV-uninfected during the last trimester of pregnancy, were eligible for study participation.

Children were scheduled for immunization with PCV7 (Prevnar®, Wyeth Vaccines, NY, USA) at 6-12 and 12-24 weeks of age, with a third dose scheduled for 38-42 weeks of age. PCV7 was given concurrently with other vaccines scheduled at the time. Other vaccines received by all children included BCG and trivalent oral polio vaccine (TOPV, OPV-Mérieux®; Sanofi-Pasteur, Lyon, France) at birth, TOPV at 6 weeks, diphtheria toxoid-tetanus-toxoid-acellular pertussis- trivalent inactivated polio vaccine and *Haemophilus influenzae* type b conjugate vaccine (DTaP-IPV//HibCV; i.e. Pentaxim®; Sanofi-Pasteur, Lyon, France) and Hepatitis B vaccine (Heberbiovac HB®, The Biovac Institute, Pinelands, South Africa) at 6-, 10-, 14-weeks of age and rotavirus vaccine (Rotarix®; GSK Biologicals, Rixensart, Belgium) at 6 and 14 weeks of age. Additionally, measles (Rouvax®, Sanofi-Pasteur, Lyon, France) vaccine was administered at 9 months of age. All vaccination was undertaken by the study staff at the study-clinic at no-cost to the participants.

The immunogenicity to PCV7 in this study was compared to immune responses in a previously enrolled cohort (N=125) of infants also born to HIV-uninfected mothers, who had received PCV7 at 6, 10 and 14 weeks. The latter cohort had been enrolled from April 2005 through to June 2006 and received their 1^st^, 2^nd^ and 3^rd^ doses of PCV7 at a mean (Standard deviation; S.D.) of 7.0 (S.D 1.0), 11.1 (S.D. 1.2) and 15.2 (S.D. 1.2) weeks of age, with anti-capsular antibody responses measured at 28-30 days after each dose as reported [10,11]. Concurrent vaccines given with PCV7 in the historical cohort included BCG and trivalent oral polio vaccine (TOPV, OPV-Mérieux®; Sanofi-Pasteur, Lyon, France) at birth; TOPV, DTPw-Hib-CV (Combact-HIB, Sanofi-Pasteur, Lyon, France) and Hepatitis B vaccine (Heberbiovac HB®, The Biovac Institute, Pinelands, South Africa) at 6, 10 and 14 weeks of age.

### Study procedures

Blood for determination of serotype specific IgG anticapsular antibodies to each of the PCV7 serotypes was obtained prior to each dose, as well as one-month (window period 3-6 weeks) following the first and second doses and 7-14 days after the third dose so as to determine anamnestic responses instead of “primary” responses following the “booster dose” of vaccine.

Samples from this study, as well as the historical-cohort, were processed at the Respiratory and Meningeal Pathogens Research Unit (RMPRU Johannesburg, South Africa) and serum archived at -70^o^C until the test were run. Determination of serum anti-capsular IgG antibody was undertaken using a standard ELISA with 22-F adsorption step as described [12]. Functional antibody activity was determined by opsonophagocytic killing assay (OPA) using differentiated HL-60 cells for serotypes 9V, 19F and 23F following the 2^nd^ and after the 3^rd^ PCV-7 dose as described [12,13].

### Statistical analysis

Data was analyzed using Stata Version 11.0 (StataCorp LP, College Station, Texas). Analysis included the anticapsular GMCs, the fold increase in GMCs and the proportion of children who developed antibody responses ≥0.35 µg/ml. The latter threshold is a putative measure of protection against invasive pneumococcal disease [14]. GMCs and 95% confidence intervals (95% C.I.) of antibody concentrations were determined following log_10_ data transformation. Opsonophagocytic geometric mean titers (GMTs) were also calculated following log_10_ transformation of the data and detectable killing activity on OPA was defined as a titer of ≥8, which was the lower threshold of the assay.

In addition, descriptive comparison were undertaken on immune responses following the second and third dose of PCV7 in the current study, compared to that observed one-month following the second and third dose of PCV7 from the historical cohort.

**Figure 1:**
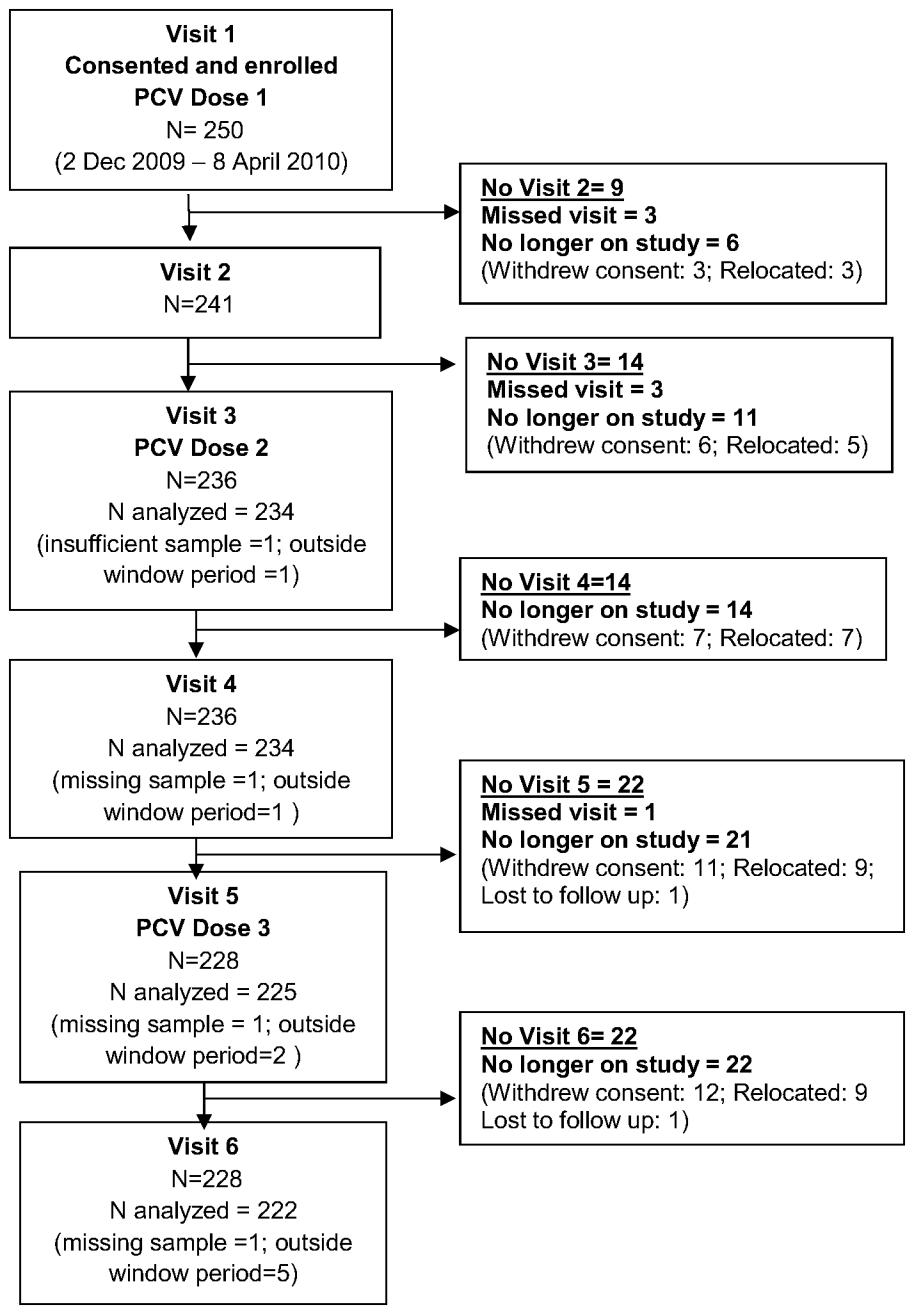
Flow Diagram of study participation. Diagram indicating number of children enrolled into the study and number excluded or lost to follow-up during the course of the study. Withdrew consent = participant no longer wished to be part of study cohort and preferred to be vaccinated at local clinic. Relocated=participant moved out of study area and therefore unable to attend study visits. Lost to follow up = study site unable to contact study participant. Outside window period = vaccination occurred outside protocol defined period of 6-12 weeks; 12-24 weeks and 38-42 weeks for the first, second and third doses respectively. PCV= 7-valent Pneumococcal Conjugate Vaccine.

### Ethics statement

The study was approved by the Human Research Ethics Committee of the University of the Witwatersrand (HREC M090824) and registered on the South African Human Research Electronic Application System (NHREC DOH-27-0511-299). Written, informed-consent was obtained from the parents of all children prior to any study-procedure.

## Results

250 Black-African children, 57% of whom were male, were enrolled and received PCV7 at mean of 6.3 (range 5.7-7.7; standard deviation [S.D.] 0.04), 15.8 (range 11.5-23.4; S.D. 0.1) and 39.9 (range 38.7-46.1; S.D. 0.05) weeks of age. The mean time interval between the first and second dose of PCV was 9.4 weeks (range 5.4-16.8 weeks; S.D. 1.4weeks), with only one child receiving the vaccines less than 8 weeks apart. Overall, 250 (100%), 235 (94.0%) and 226 (90.4%) received their first, second and third doses of PCV7 within the protocol defined periods. Analysis at any specific time-point was limited to those children who had complied with earlier protocol-defined time-points, including up to the analyzed time-point. Overall, 228 (91.2%) participants were active on study by the time of the last analyzed time-point (Figure 1).

**Figure 2:**
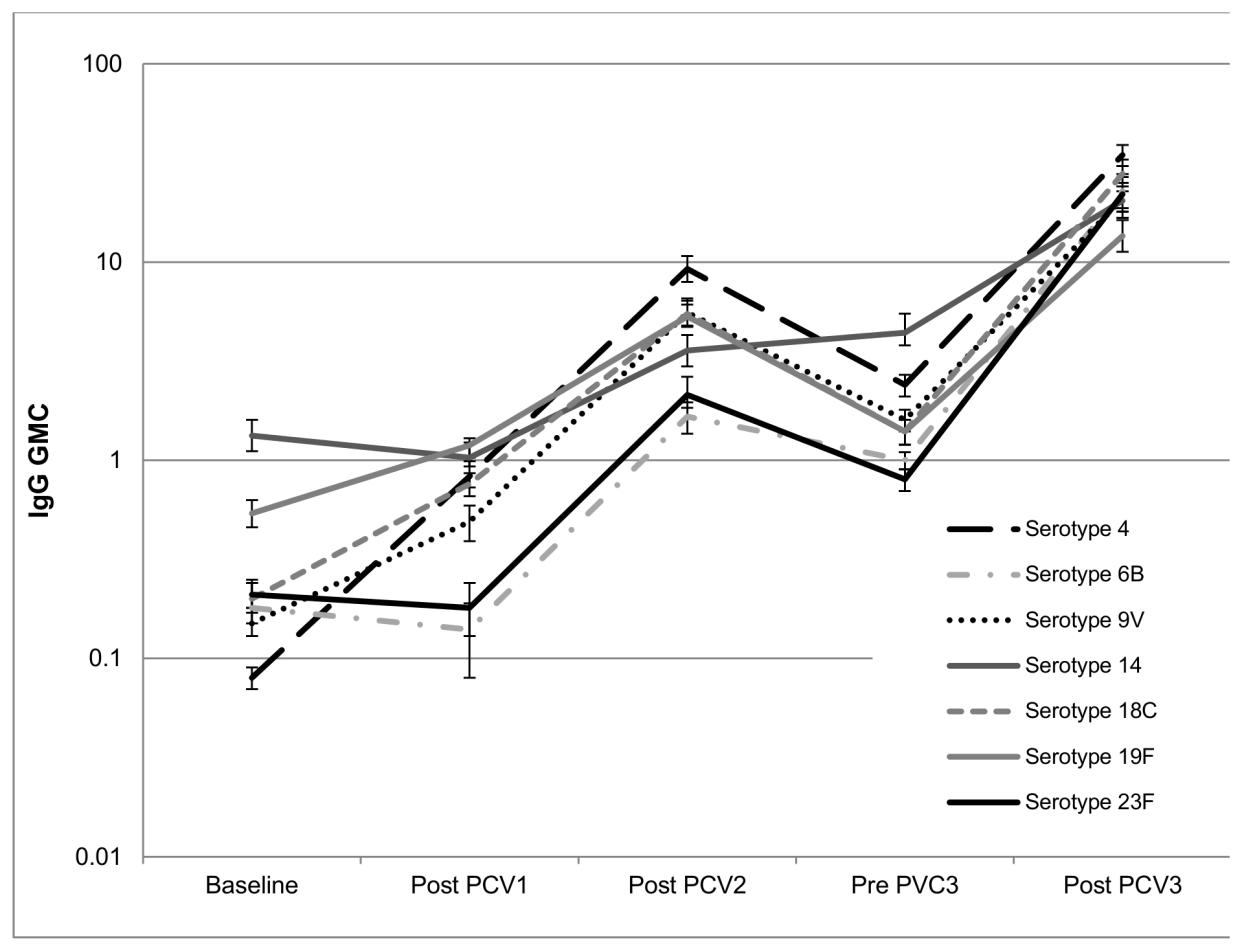
IgG GMC following a 2-dose primary series and a booster dose of PCV-7. IgG antibody GMC for each of the 7 serotypes following vaccination with PCV-7, administered as a 2-dose primary series, given at 6- and 14-weeks of age, and a booster dose at 40 weeks of age. GMC were measured at baseline, after the first dose (Post PCV1) and second dose (Post PCV2) and also prior to (Pre PCV3) and after (Post PCV3) the booster dose. GMC = Geometric Mean Concentrations, PCV-7= 7-valent Pneumococcal Conjugate Vaccine.

### Quantitative and qualitative antibody responses

The vaccine-serotype specific GMCs following each PCV7 dose is shown in Table S1 and Figure 2. There was a 3.48 (serotype 14) to 11.73 (serotype 23F) fold increase in GMCs between the first and second PCV7 doses, which was consistently higher for all serotypes compared to the fold increase between baseline and after the first PCV7 dose; Table S1. The GMCs to all serotypes declined by age of 39.9 weeks when the 3^rd^ PCV7 dose was administered, which was followed by a 4.74 (serotype 14) to 29.25 (serotype 23F) fold- increase in GMC following the 3^rd^ PCV7-dose; Table S1 and Figure 2.

The proportion of infants with serotype-specific antibody ≥0.35 µg/ml following the first PCV7-dose ranged from 20.6–21.9% (serotypes 23F and 6B) to 94.0% for other serotypes. This increased to 84.1% and 89.3% for serotypes 6B and 23F, respectively, and ≥97.4% for other serotypes following the 2^nd^ PCV7-dose; Table S1. Although GMCs declined, persistence of antibody ≥0.35 µg/ml remained similar to after the 2^nd^ PCV7-dose when evaluated just prior to the third PCV7-dose; Table S1. Following the 3^rd^ PCV7-dose, >96.8% of children had antibody concentrations of ≥0.35 µg/ml to each serotype. The proportion of children who had ≥4 fold increase in GMCs following the 3^rd^ PCV-dose ranged between 61.8% for serotype 14 to 92.7% for serotype 18C; Table S1.

The proportion of children with OPA ≥8 to 9V, 19F and 23F were 78.5%, 69.4% and 71.2%, following the 2^nd^ PCV7-dose, which increased significantly to 93.6% (p<0.001), 77.6, % (p<0.001), and 89.7% (p<0.001), respectively, after the third PCV7-dose; Table 1.

**Table 1:** Opsonophagocytic activity assay following the second and third doses of 7-valent pneumococcal conjugate vaccine for 3 different pneumococcal serotypes.

	OPA titer ≥8 (95%CI)		OPA geometric mean titer (95%CI)	
	Post-dose 2 N; % (95%) CI	Post-dose 3 N; % (95% CI)	Post-dose 2 GMT (95% CI)	Post-dose 3 GMT (95% CI)
Serotype 9V	168/214; 78.5% (72.4-83.8)	161/172; 93.6% (88.8-96.8)	22 (19-26)	79 (66-95)
Serotype 19F	154/221; 69.4% (62.8-75.3)	135/174; 77.6% (70.6-83.5)	19 (16-22)	47 (35-61)
Serotype 23F	161/225; 71.2% (64.8-77.0)	156/174; 89.7% (84.1-93.7)	30 (24-37)	137 (103-182)

OPA: Opsonophagocytic activity assay, 95% CI = 95% confidence interval, GMT: Geometric Mean Titres

### Comparison to historical cohort receiving PCV7 at 6, 10 and 14 weeks of age

**Figure 3:**
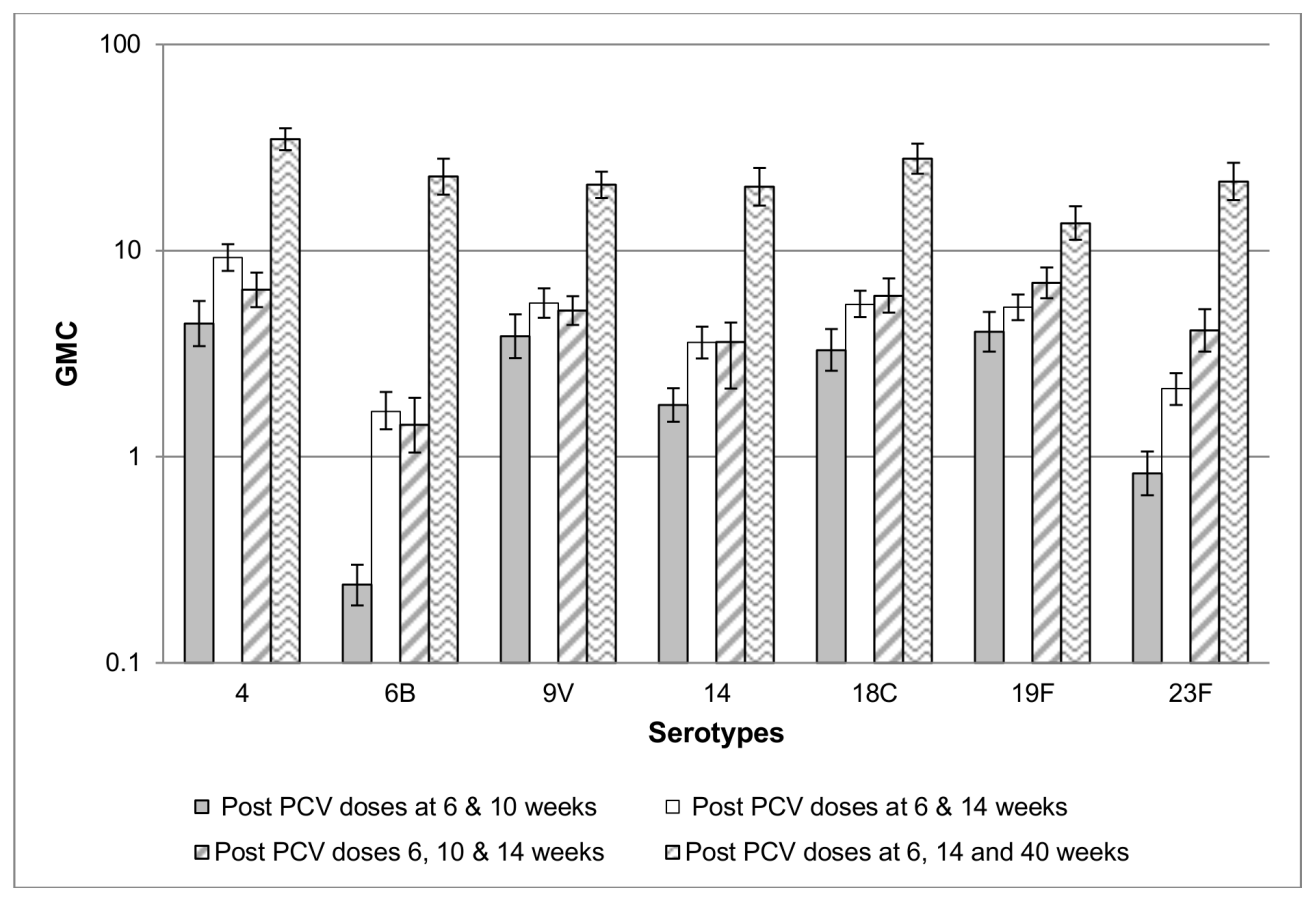
Comparison of IgG GMC following 2 and 3 doses of PCV-7 administered at different intervals. Comparison of IgG antibody GMC following vaccination with PCV-7 administered either at 6-;10- and 14 weeks of age or at 6 and 14 weeks of age and a booster at 40 weeks of age. GMCs were assessed after the second and after the 3rd dose in the series. GMC = Geometric Mean Concentrations, PCV-7= 7-valent Pneumococcal Conjugate Vaccine, IgG = Immunoglobulin G.

Compared to the historical cohort who received PCV7 at mean ages of 7.0, 11.1 and 15.2 weeks, GMCs were higher for most serotypes in the current cohort when comparing immune responses following the second PCV7-dose; albeit overlap of the 95% CI for serotypes 9V and 19F; Figure 3. GMCs in this study-cohort were generally similar (serotypes 6B, 9V, 14, 18C, 19F) except for serotypes 4 (higher) and 23F (lower) ) following the 2^nd^-PCV7 dose in the current-cohort compared to one-month after the third-PCV7 dose in the historical comparator-group; Figure 3 and Table 2. On comparison of immune responses after the third PCV7 dose in the current compared to the historical cohort, GMCs were general 4-5 fold greater in the current cohort to most serotypes (4, 9V, 14, 18C, 23F) and 16.3-fold greater for serotype 6B; Figure 3 and Table 2.

**Table 2:** IgG anti-capsular geometric mean concentrations following a second dose given at a 4 week or 8 week interval and a third dose of pneumococcal conjugate vaccine.

Group	Historical Cohort*	Study Cohort**	Historical Cohort	Study Cohort	
Serotype	Post dose 2 (4 week interval)	Post dose 2 (8 week interval)	Post dose 3	Post dose 3	Fold diff 3 vs. 3***
	n= 116 GMC (95% CI)	n= 234 GMC (95% CI)	n= 114 GMC (95% CI)	N= 222 GMC (95% CI)	
4	4.43 (3.44-5.70)	9.23 (7.96-10.70)	6.45 (5.32-7.82)	34.63 (30.63-39.14)	5.37
6B	0.24 (0.19-0.30)	1.66 (1.36-2.03)	1.43 (1.05-1.94)	22.82 (18.67-27.89)	16.30
9V	3.84 (3.01-4.91)	5.56 (4.73-6.52)	5.12 (4.36-6.0)	20.83 (17.99-24.11)	4.06
14	1.78 (1.48-2.15)	3.58 (3.00-4.28)	3.6 (2.90-4.47)	20.40 (16.52-25.19)	5.66
18C	3.29 (2.61-4.15)	5.48 (4.75-6.33)	6.02 (5.0-7.25)	27.87 (23.53-33.0)	4.62
19F	4.04 (3.24-5.03)	5.31 (4.60-6.13)	6.97 (5.88-8.27)	13.57 (11.21-16.42)	1.95
23F	0.83 (0.65-1.06)	2.14 (1.78-2.56)	4.09 (3.23-5.19)	21.99; (17.93-26.98)	5.37

GMC = Geometric Mean Concentration

* Historical cohort: vaccinated at 6-10-14 weeks

** Study cohort: vaccinated at 6-14-40 weeks

*** Comparing the fold increase in GMC post dose 3 for the 2 different cohorts

**Figure 4:**
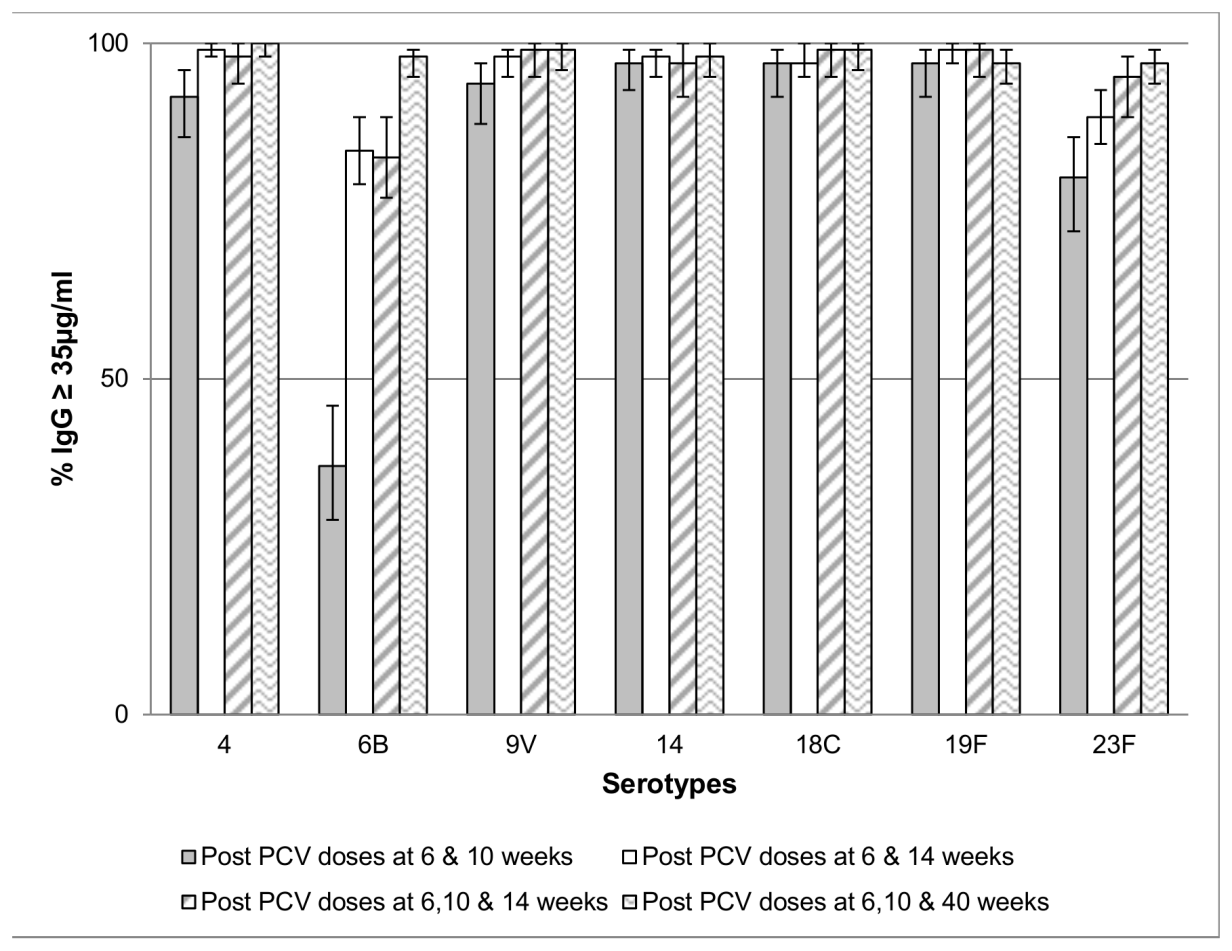
Proportion of infants with IgG concentrations of ≥ 35µg/ml following 2 and 3 doses of PCV-7. The proportion of infants with serum anti-capsular IgG antibody concentration ≥ 35µg/ml, a putative measure of protection against invasive pneumococcal disease, following vaccination with PCV-7. Vaccine was administered at 6,10 and 14 weeks of age or at 6,14 and 40 weeks of age. Antibody concentrations were assessed after the second and after the third dose in the series. PCV-7= 7-valent Pneumococcal Conjugate Vaccine, IgG = Immunoglobulin G.

On comparison of the proportion of infants with antibody concentration ≥0.35 µg/ml between the current and historical cohorts, a higher proportion of children in the current cohort had antibody ≥0.35 µg/ml to serotype 6B (84.1% vs. 37.0%) and a similar trend was observed for serotype 23F (89.3% vs. 80.2%) following the second doses of PCV7; Figure 4. Also, the proportion of children with antibody ≥0.35 µg/ml was almost identical following the second-dose in the current cohort compared to after the third dose in the historical cohort; Figure 4.

## Discussion

The findings of our study indicate robust quantitative and qualitative antibody responses of a PCV7 dosing schedule when administered at 6, 14 and 40 weeks of age. The immune responses of this dosing-schedule elicited similar concentrations of antibody levels following the 2^nd^ PCV7-dose compared to after the 3^rd^ PCV7-dose in a historical cohort who were vaccinated at 6-10-14 weeks of age. This suggests that the current South African PCV7 dosing schedule would be as effective after the second PCV7-dose compared to after the third PCV7 dose when administered at 6-10-14 weeks, which had previously been shown to protect against invasive pneumococcal disease and pneumonia in the same setting[15]. Notably, the immune responses following the 3^rd^ PCV7-dose at 40 weeks of age in our study were associated with higher quantitative antibody responses, compared to after the 3^rd^-PCV7 dose at 14 weeks of age in the historical-cohort. Although there was no difference in the proportion of infants with antibody concentrations ≥0.35 µg/ml after the 3^rd^ PCV7-doses, except for being higher for serotype 6B in the current compared to the historical cohort, the higher GMCs in the 6-14-40 week dosing schedule may have a bearing on higher persistence of antibody into later life, which may consequently affect the durability of protection against pneumococcal disease. Particularly, such higher GMCs may confer greater protecting against pneumococcal mucosal infections such as acute otitis media and pneumonia, should protection against mucosal infections require higher levels of antibody compared to that required for protection against invasive pneumococcal disease[4,16].

When deciding on PCV dosing schedules, particularly when choosing between a 2+1 and 3+0 schedule, the WHO recommends that countries should consider epidemiology, coverage and timeliness of vaccination. One concern for low income countries is that a three dose schedule may compromise protection due to insufficient immune responses after 2^nd^ dose. Goldblatt et al. reported that immune responses to serotype 6B and 23 F remained poor, even when given at 2 and 4 months with concomitant DTaP5/IPV/Hib-TT, with only 47% and 62% reaching IgG levels of ≥0.35µg/ml, respectively. Reduced efficacy for serotype 6B in the UK was considered to be possibly related to this poor immune response and the WHO position paper states that a 2+1 schedule might not offer optional individual protection for 6B and 23F. In our study, immune responses to all seven serotypes, including 6B and 23F, were comparable following the second PCV7 dose compared to after the third PCV7 dose in the 6-10-14 week schedule.

In the study by Goldblatt et al. [4], immune responses to serotypes 6B, 14F and 23F were significantly lower following two-doses of PCV7 given one-month apart compared to when spaced at least two months apart. The results from our study corroborate the finding of better immune responses when PCV7 is spaced two months apart, for the proportion of infants attaining antibody levels ≥0.35µg/ml, as well as in relation to GMC levels following the second PCV7-dose. Although the study design allowed for wide ranges in the age for immunization, the mean age and small standard deviation of time when vaccines were administered indicated that the majority of children received their vaccines close to the time when vaccination was planned for in the immunization program. The heightened immunogenicity of spacing PCV7 at least two months apart, was particularly notable for immune responses to serotype 6B, which is recognized to be less immunogenic than other PCV7-serotypes, and without having compromised the immunogenicity to other serotypes. Protection against serotype 6B is especially important in settings such as South Africa, where 67% of invasive pneumococcal disease (IPD) in the <5 year age group in the pre-vaccine era was due to PCV7 -serotypes, of which 30% were due to 6B[17].

Although the observation of higher IgG GMCs being induced when the 3^rd^ dose was given at 9 months compared to at 15 weeks of age, a limitation of this observation was that antibody responses were measured 2 weeks after 3^rd^ dose in this study compared to 4 weeks after 3^rd^ dose in the historical cohort. While this may bias the results of showing higher antibody concentrations in the current cohort, the magnitude of difference as well as the fold-increase in antibody concentrations compared to after the third dose in the historical-cohort suggest these differences likely to be true.

Other limitations of our study include it not being a randomized study comparing of the two dosing schedules, however, the historical cohort of children were primarily from the same community. Also, the pneumococcal antibody assays were done using a standardized method with a standard reference serum and in the same laboratory, which in part mitigates this limitation. We were, however, unable to undertake a comparison of OPA responses for the current study-cohort compared to the historical-cohort [14], since the OPA assay may be affected by variation due in the batches of reagents, cell lines and target bacterial strains overtime. In addition, this investigator-sponsored study was limited by resources and consequently only undertook OPA evaluation for three of the seven serotypes. This, however, included serotypes with inherent differences in their immunogenicity, including the relatively poorly immunogenic serotype 23F and more immunogenic serotypes 9V and 19F. A further limitation of our study was the change from whole-cell pertussis combination vaccines used in the historical cohort to acellular pertussis containing pentavalent vaccine (Pentaxim™) as concomitantly administered vaccines. The concomitant administration of acellular-pertussis instead of whole cell pertussis containing vaccines have, however, been associated with dampening the immune responses to protein-polysaccharide conjugate vaccines, including in immunogenicity studies involving *Haemophilus influenzae* type b conjugate vaccine and pneumococcus tetanus-toxoid conjugate vaccines[18,19]. Other studies have shown that PCV7 was highly immunogenic when concomitantly administered with DTaP–IPV-Hib and DTaP–IPV–HBV/Hib combinations and that there were no clinically significant interactions or influence in the immunogenicity [20–22]. The robust immune responses in the current study cohort who received acellular-pertussis containing vaccines are therefore suggestive of absence of interference of immune responses to PCV in African children and when used as in the current South African schedule.

A major reason for adopting the current dosing schedule in South Africa, related to conferring greater durability of protection against invasive pneumococcal disease in HIV-infected children. Although our study was not designed to evaluate the immune responses of this dosing schedule in HIV-infected children, the similarity in immune responses following each of the three doses of PCV7 at 6-10-14 weeks between HIV-infected managed early-on with anti-retroviral therapy, as is currently the practice in South Africa, compared to HIV-uninfected children [23], suggest that the immunogenicity of the current South African dosing schedule is likely to perform similarly in HIV-infected children initiated early on anti-retroviral treatment. The effectiveness of the 6-14-40 week dosing schedule in South Africa is currently being evaluated in case-control studies against invasive pneumococcal disease and pneumonia[24,25].

## Supporting Information

Table S1 **IgG serotype anticapsular antibody geometric mean concentration and proportion of infants with antibody ≥0.35 µg/ml following the first, second and 3^rd^ doses of 7-valent pneumococcal vaccine.**
(DOCX)
