# Supplementary material for: Immunogenicity of Seven-Valent Pneumococcal Conjugate Vaccine Administered at 6, 14 and 40 Weeks of Age in South African Infants
Source: PLoS One. 2013 Aug 28;8(8):e72794. doi: 10.1371/journal.pone.0072794 (PMC3755982; doi:10.1371/journal.pone.0072794)
Supplement: Table S1 — IgG serotype anticapsular antibody geometric mean concentration and proportion of infants with antibody ≥0.35 µg/ml following the first, second and 3rd doses of 7-valent pneumococcal vaccine. (DOCX) [file pone.0072794.s001.docx]

**Table S1.** IgG serotype anticapsular antibody geometric mean concentration and proportion of infants with antibody ≥0.35 µg/ml following the first second and 3^rd^ doses of 7-valent pneumococcal vaccine.

| Study visit | Antibody. measure | Serotype | | | | | | |
| --- | --- | --- | --- | --- | --- | --- | --- | --- |
|  |  | 4 | 6B | 9V | 14 | 18C | 19F | 23F |
| Pre- vaccine N=250 | N (%; 95%CI) >0.35 µg/ml | 30 (12.0; 8.2-16,6) | 69 (27.6; 22.5-33.5) | 70 (28.0; 22.5-34.0) | 207 (82.0; 77.5-87.2) | 91 (36.4; 30.4-42.6) | 165 (66.0; 59,7-71.8) | 91 (36,4; 30.4-42.6) |
|  | GMC (95%CI) | 0.08 (0.07-0.09) | 0.18 (0.15-0.21) | 0.15 (0.13-0.18) | 1.33.(1.11-1.60) | 0.20 (0.17-1.24) | 0.54 (0.46-0.63) | 0.21 (0.18-0.25) |
| Post Dose 1 N= 234 | N (%; 95% CI) >0.35 µg/ml | 191 (81.6; 76.0-86.3) | 51 (21.9; 16.6-27.6) | 140 (59.8; 53.2-66.1) | 209 (89.3; 84.6-92.9) | 182 (77.8; 71.9-82.9) | 220 (94.0; 90.1-96.6) | 48 (20.6; 16.6-27.6) |
|  | GMC (95%CI) | 0.83 (0.72-0.96) | 0.14 (0.12-0.17) | 0.49 (0.43-0.57) | 1.03 (0.91-1.16) | 0.76 (0.65-0.88) | 1.19 (1.07-1.32) | 0.18 (0.16-0.20) |
|  | GMC fold increase vs. baseline (95%CI) | 10.21 (8.28-12.59) | 0.79 (0.66-0.96) | 3.2 (2.57-3.98) | 0.78 (0.65-0.94) | 3.51 (2.78-4.41) | 2.27 (1.85-2.78) | 0.85 (0.72-1.00) |
| Post Dose 2 N= 234 | N (%; 95%CI) >0.35 µg/ml | 233 (99.5; 97.6-99.9) | 197 (84.1; 78.8-88.6) | 228 (97.8; 95.0-99.2) | 229 (97.8; 95.0-99.3) | 228 (97.4; 94.5-99.0) | 233 (99.5; 97.6-99.9) | 209 (89.3; 84.6-92.9) |
|  | GMC (95%CI) | 9.23; (7.96-10.70) | 1.66; (1.36-2.03) | 5.56; (4.73-6.52) | 3.58; (3.00-4.28) | 5.48; (4.75-6.33) | 5.31; (4.60-6.13) | 2.14; (1.78-2.56) |
|  | GMC fold increase vs. pre-dose 2 (95%CI) | 11.3 (9.67-13.24) | 11.5 (9.19-14.44) | 11.32 (9.45-13.55) | 3.48 (2.83-4.27) | 7.3 (6.33-8.83) | 4.55 (3.93-5.28) | 11.73 (9.42-14.61) |
| Pre-Dose 3 N= 225 | N (%; 95%CI) >0.35 µg/ml | 220 (97.7; 94.8-99.2) | 190 (84.4; 79.0-88.9) | 208 (92.4; 88.1-95.5) | 221 (98.2; 95.5-99.5) | 213 (94.6; 90.8-97.2) | 207 (92.0; 87.6-95.1) | 167 (74.2; 67.9-79.8) |
| Post-booster Dose 3 N=222 | N (%; 95%CI) >0.35 µg/ml | 222 (100.0; 98.3-100.0) | 217 (97.7; 94.8-99.2) | 219 (98.6; 96.1-99.7) | 218 (98.2; 95.4-99.5) | 219 (98.6; 96.1-99.7) | 215 (96.8; 93.6-98.7) | 215 (96.8; 93.6-98.7) |
|  | GMC (95%CI) | 34.63; 30.63-39.14) | 22.82; 18.67-27.89) | 20.83; (17.99-24.11) | 20.40; (16.52-25.19) | 27.87; (23.53-33.0) | 13.57; (11.21-16.42) | 21.99; (17.93-26.98) |
|  | GMC fold increase vs. pre-dose 3 (95%CI) | 14.8 (12.8-17.0) | 23.5 (19.51-28.46) | 13.52 (11.7-15.6) | 4.74 (3.90-5.77) | 20.3 (17.26-24.01) | 9.89 (8.21-11.90) | 29.25 (23.75-36.03) |

GMC = Geometric Mean Concentration; 95% CI= 95% Confidence Interval
